# Ultrathin hydrophobic films based on the metal organic framework UiO-66-COOH(Zr)

**DOI:** 10.3762/bjnano.10.65

**Published:** 2019-03-06

**Authors:** Miguel A Andrés, Clemence Sicard, Christian Serre, Olivier Roubeau, Ignacio Gascón

**Affiliations:** 1 Departamento de Química Física and Instituto de Nanociencia de Aragón (INA), Universidad de Zaragoza, 50009 Zaragoza, Spain; 2 Instituto de Ciencia de Materiales de Aragón (ICMA), CSIC and Universidad de Zaragoza, 50009 Zaragoza, Spain; 3 Institut Lavoisier de Versailles, UVSQ, CNRS, Université Paris-Saclay, 45 avenue de États-Unis, 78035 Versailles Cedex, France; 4 Institut des Matériaux Poreux de Paris, FRE 2000 CNRS Ecole Normale Supérieure de Paris, Ecole Supérieure de Physique et de Chimie Industrielles de Paris, PSL Research University, 75005 Paris, France

**Keywords:** hydrophobic coating, Langmuir–Blodgett (LB) films, metal organic framework (MOF), surface modification, UiO-66-COOH(Zr)

## Abstract

This work reports on the fabrication, optimization and characterization of ultrathin films containing submicrometer particles (sMPs) of the hydrophilic and water stable UiO-66-COOH(Zr) metal organic framework (MOF). MOF particles of ≈200 nm have been synthesized and assembled at the air–water interface by the Langmuir–Blodgett technique. The use of different solvents, mixtures of solvents and surfactants has been investigated in order to improve the stability of MOF dispersions and reduce particle aggregation. The compact MOF/surfactant films containing 10 wt % octadecylphoshonic acid (ODP) have been deposited on substrates of different nature by Langmuir–Blodgett (LB) and Langmuir–Schaefer (LS) methods, showing that the presence of even only one MOF/ODP monolayer can increase the water contact angle of highly hydrophilic substrates such as mica or glass up to 120°. These films were characterized by scanning electron microscopy, grazing incidence X-ray diffraction, Fourier transform infrared spectroscopy and atomic force microscopy, revealing the formation of a continuous film where ODP molecules adopt an almost vertical position and cover MOF particles. Moreover, the presence of MOF particles significantly enhances the surface roughness and allows ultrathin, hydrophobic coverage to be obtained. Finally, it has been shown that the crystallinity and the porosity of the MOF remains almost unaltered in MOF/ODP films.

## Introduction

Metal organic frameworks (MOFs) are well-known, crystalline, porous materials formed by metal ions (or metallic clusters) and organic ligands coordinated in a pre-designed manner to form pores and/or channels of desired dimensions [1]. These materials tend to have a very high surface area and present several advantages compared to traditional porous inorganic materials, including chemical diversity and good compatibility with polymers and surfactants [2–3]. Moreover, several strategies which allow the introduction of different functional units into a single framework in a combinatorial fashion have been applied for MOF post-synthetic modification [4] in order tune and optimize MOF properties. All these features make MOFs very attractive for a wide variety of applications [5], including gas storage [6], membranes for separation processes [7], heterogeneous catalysis [8], sensing [9] or drug delivery [10], among others. Many of these applications require the formation of MOF films onto different kinds of surfaces with precise control of film thickness and homogeneity [11]. Therefore, several strategies have been used for the deposition of MOF films [12], including direct growth [13–14], electrochemical deposition [15], inkjet-printing [16], dip-coating [17], layer-by-layer [18–20], Langmuir–Blodgett [21–22], chemical vapor deposition [23], spin-coating [24] and spray methods [25].

Compared to other methodologies that allow the deposition of MOF thin films, the Langmuir–Blodgett (LB) technique presents some advantages. Previous functionalization of the surface is not necessary and the film obtained can be as thin as one monolayer of MOF particles, which is especially interesting for the development of MOF-based devices that require the use of very small MOF quantities. In some recent studies, we have reported the fabrication at the air–water interface of dense monolayers of nanoparticles of MIL-101(Cr) and MIL-96(Al) MOFs that can be transferred onto different kinds of substrates using the Langmuir–Blodgett (LB) or Langmuir–Schaefer (LS) deposition methods.

Furthermore, the use of these films for CO_2_ sensing [21,26] or organic solvent nanofiltration [27] has been investigated. Additionally, we have also explored the fabrication of mixed LB films containing nanometric or micrometric particles of the MOF NH_2_-MIL88B(Fe) and a commercial polyimide, showing that it is possible to obtain ultrathin MOF–polymer hybrid films with a homogeneous distribution of MOF particles within the polymer matrix [28].

In this contribution, submicrometer particles (sMPs) of the metal organic framework UiO-66-COOH(Zr) with size 200 ± 80 nm have been synthesized and the formation of MOF films at the air–water interface has been studied. This MOF was selected due to its chemical stability, its environmentally friendly aqueous synthesis route that leads to submicrometer particles, and its good CO_2_ adsorption capacity altogether maintaining good water stability [29–31]. In addition, our experience on the fabrication of MOF LB films has shown that hydrophilic MOFs can lead to LB films of good quality (e.g., MIL-101(Cr) [21] and MIL-96(Al) [26]) and UiO-66-COOH(Zr) with free pendant –COOH groups also fulfills this requirement.

Firstly, the effect of using different solvents (or mixtures of solvents) for the preparation of MOF dispersions has been investigated. Additionally, different amounts of two well-known surfactants that form stable and compact Langmuir films, behenic acid (BA) [32] or octadecylphosphonic acid (ODP) [33], have been added to MOF suspensions in order to improve their stability and reduce particle aggregation, proving that the addition of a 10% in mass of ODP (relative to MOF mass) significantly improves the suspension stability and film homogeneity.

Interestingly, a recent study has shown that alkyl phosphonic acids, such as ODP, interact with the zirconium oxide clusters situated near and on the surface of Zr-based MOFs and the surface free energy on the exterior of the MOF can be reduced by the octadecyl alkyl chains, spawning superhydrophobic materials [34]. The use of different strategies to modify the MOF surface in order to enhance its water stability has been reported in the last years. This was necessary as several MOFs show poor stability in water-containing environments that hinder their use in many real-world applications [34–35]. Superhydrophobic MOFs could be of interest for a great variety of technological applications, including coatings, paints and fabrics [36]. Moreover, it has been shown [37] that these materials could be used as catalysts and in gas separation under humid conditions.

In this contribution, mixed OPD/MOF ultrathin films have been fabricated onto glass, calcium fluoride, quartz crystal microbalance (QCM), Si(100) substrates and mica and characterized using scanning electron microscopy (SEM), Fourier transform infrared spectroscopy (FTIR), X-ray diffraction (XRD), atomic force microscopy (AFM) and water contact angle (WCA) measurements. The results obtained demonstrate the hydrophobic character of the films obtained, since a single mixed ODP/MOF LB film can increase the water contact angle of highly hydrophilic substrates (glass and mica) up to 120° while maintaining a high transparency.

## Results and Discussion

### Optimization of MOF submicrometer particle Langmuir films: solvent mixtures and surfactants

Spherical UiO-66-COOH(Zr) submicrometer particles (sMPs) of ≈200 nm diameter were synthesized and characterized (see Experimental section and Supporting Information File 1, Figure S1 for PXRD pattern, SEM image and N_2_ sorption isotherms at 77 K). Langmuir films of pure MOF sMPs obtained at the air–water interface using diluted suspensions in chloroform showed a lack of reproducibility, which could be ascribed to poor suspension stability, mostly associated with particle aggregation (see Supporting Information File 1 for details). In order to improve the suspension stability and reduce the particle aggregation, the use of different mixtures chloroform/solvent (short chain alcohols or THF) was explored and THF was eventually chosen (see Supporting Information File 1 for details), as previously done for Langmuir film fabrication of other materials [38–40]. However, although the suspension stability was clearly improved using a 1:4 THF/CHCl_3_ volume ratio, the reproducibility of the π–*A* isotherms was not favorable. This result motivated us to seek other means to improve the MOF sMPs suspension, while avoiding material loss into the subphase. The use of an auxiliary surfactant was chosen since previous studies [21,40–42] have shown that it is an efficient method to obtain stable Langmuir films. When binary mixtures MOF/surfactant are used, the surfactant is expected both to reduce the particle aggregation and improve the formation of compact films at the air–water interface. In this approach, an appropriate surfactant should first be chosen (i.e., it should not react with the MOF). Second, its concentration has to be optimized to enhance film formation in order to preserve the MOF properties. In this study, molecular surfactants were used because of solubility issues of charged surfactants in chloroform.

Mixtures MOF/behenic acid (BA) have been already used with success for MIL-101(Cr) [21]. In this work, MOF suspensions with BA surfactant concentrations in the range 1–10 wt % were tested. Due to the low MOF amounts used, a BA concentration of less than 5 wt % was insufficient for the formation of a BA Langmuir film. For higher BA concentrations, the normalized surface pressure/area isotherms showed lift-off at lower areas per MOF mass in comparison to the bare UiO-66-COOH(Zr) suspensions. Moreover, from the SEM analysis of drop-cast samples from these mixtures, some particles seemed to be fused which could suggest a side reaction between the MOF and the behenic acid (Supporting Information File 1, Figure S5).

Then, octadecylphosphonic acid (ODP) was explored as an alternative, with a polar phosphonic acid head group instead of the carboxylic acid function. Dispersibility studies were performed first in view of preparing more concentrated MOF suspensions. From these studies, a great improvement in the MOF dispersibility was observed when the suspension contained ODP. The optimal amount of ODP was estimated to be close to 0.8 µmol·mL^−1^. Different MOF/ODP suspensions in the MOF concentration range 0.025–0.11 mg·mL^−1^ containing the same ODP concentration (0.8 µmol ODP·mL^−1^) were used for the formation of Langmuir films.

**Figure 1:**
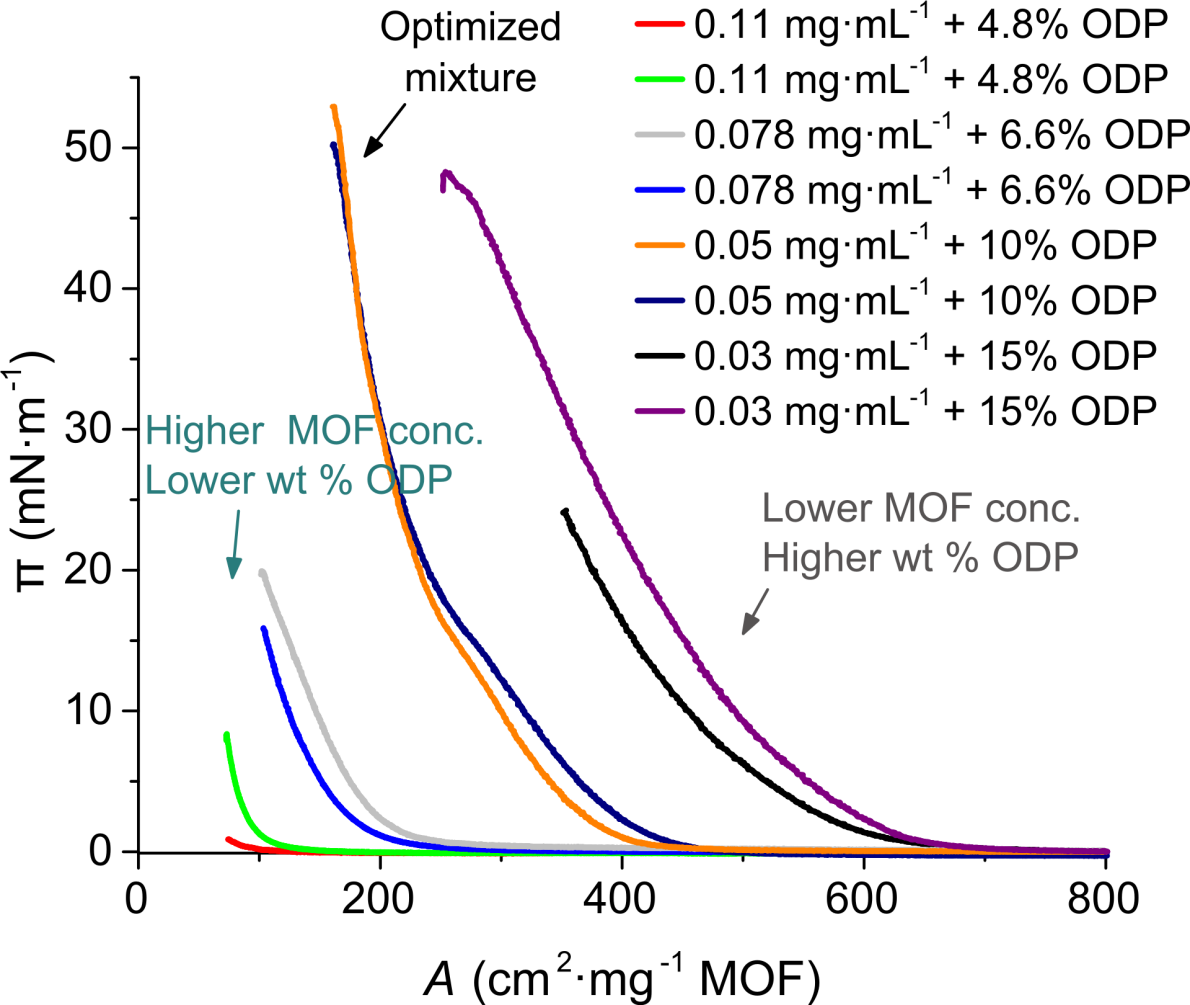
Surface pressure–area (π–*A*) isotherms obtained using different UiO-66-COOH(Zr) + ODP suspensions in the MOF concentration range 0.025–0.11 mg·mL^−1^. ODP content in all suspensions is 0.8 µmol·mL^−1^. Note that relative ODP wt % changes because the mass amount of ODP for all the dispersions is the same but MOF concentration changes.

Figure 1 shows surface pressure/area isotherms for these mixtures. In this case, an expansion of the isotherms to higher areas per MOF mass was observed in comparison to the isotherms of the pure UiO-66-COOH(Zr) sMPs. Moreover, a better reproducibility in the assayed concentration range (compared to pure MOF films) is observed upon addition of ODP (every run at each surfactant concentration was repeated twice). Brewster angle microscopy (BAM) images together with SEM inspection of preliminary Langmuir film transfers showed that the best results were obtained for the 0.05 mg MOF·mL^−1^ + 10 wt % ODP suspensions. Moreover, these suspensions led to the best reproducibility in terms of Langmuir film formation (see Figure 1).

**Figure 2:**
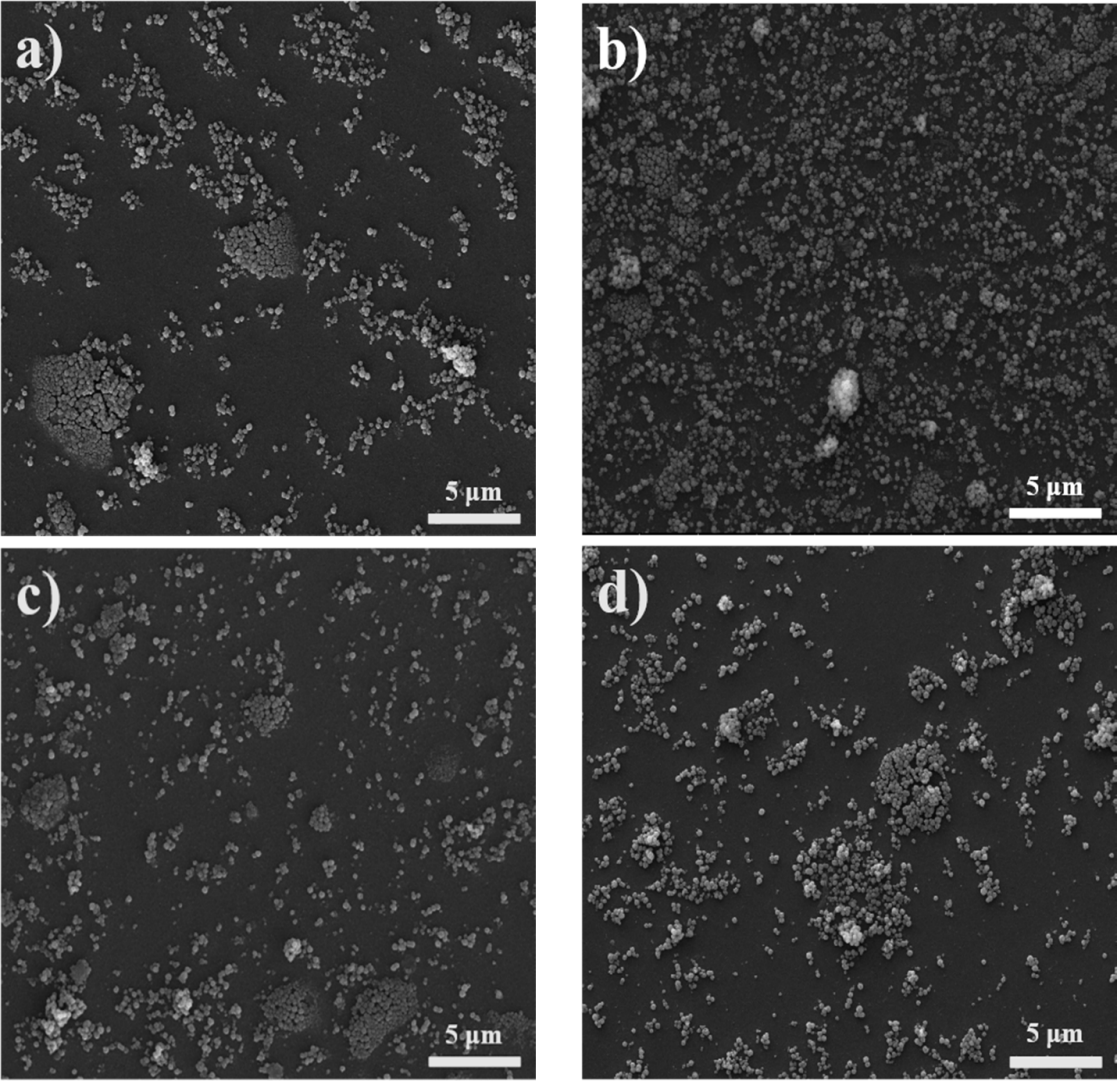
SEM images of Langmuir–Blodgett films transferred at: (a) 25 mN·m^−1^, (b) 30 mN·m^−1^, (c) 35 mN·m^−1^ and (d) 45 mN·m^−1^. Spreading suspensions were 0.05 mg·mL^−1^ UiO-66-COOH(Zr) + 10 wt % ODP mixtures. The scale bars correspond to 5 µm.

When these surface pressure–area isotherms are normalized versus the area per ODP molecule, the lift-off always appears at lower areas than in pure ODP films. This reflects that not all the ODP molecules are in contact with water, suggesting that a certain amount of the surfactant should be adsorbed at the surface of the MOF particles. To confirm this fact, and to analyze the architecture of the films, Langmuir films prepared with the optimal MOF/surfactant concentration were transferred onto glass substrates at different surface pressures. Figure 2 shows SEM images of these Langmuir–Blodgett films. From the analysis of the SEM images, it appears that 30 mN·m^−1^ is the optimal transfer pressure in the sense of a more homogeneous MOF distribution and lower particle aggregation. Pressure values higher than 30 mN·m^−1^ lead to a worse MOF coverage of the surface together with a higher particle aggregation, probably due to the collapse of the ODP subjacent film.

LS and reverse Langmuir–Schaefer (RLS) transfers were also conducted to check if some material loss occurred during the LB transfer process. Similar results were obtained with RLS while conventional Langmuir–Schaefer transfers led to greater particle aggregation above 30 mN·m^−1^. In addition, conventional glass substrates were functionalized to make them hydrophobic (by previous self-assembly of the hydrophobic silane HMDS) or more hydrophilic (prior transfer of 4 layers of behenic acid onto the substrate). However, no improvement was observed in any case.

In view of the results obtained, new MOF/ODP mixtures were prepared with a fixed amount of ODP (10 wt %) in the MOF concentration range of 0.03–0.11 mg·mL^−1^. Langmuir films were fabricated using these mixtures and surface pressure–area and surface potential–area isotherms were registered. The most diluted suspensions (0.03 mg·mL^−1^ UiO-66-COOH(Zr)) led to poor reproducibility, whereas the isotherms of the more concentrated ones were acceptable. To compare the effect of increasing the ODP content on the more concentrated suspensions, π–*A* isotherms were compared to those of the mixtures containing a variable wt % content of ODP (Supporting Information File 1, Figure S6). An expansion on the lift-off areas was observed and higher pressures were reached. From the SEM analysis of transferred films, the particle density and film homogeneity was not better than that corresponding to the 0.05 mg·mL^−1^ MOF + 10 wt % ODP mixture. Moreover, the most diluted suspensions showed significant aggregation, which probably led to the poor reproducibility of the π–*A* isotherms.

Overall, LB films of good quality made of UiO-66-COOH(Zr) sMPs can be fabricated by optimizing the composition of the dispersion which involves the use of ODP as a surfactant. We found that the optimal conditions are achieved using chloroform suspensions containing 0.05 mg·mL^−1^ of UiO-66-COOH(Zr) and 10 wt % of ODP. Consequently, this suspension was used to prepare the films that were further characterized.

### CO_2_ adsorption studies

In order to study the effect of the surfactant on the adsorption capacity of the MOF sMPs, CO_2_ adsorption studies were performed using the quartz crystal microbalance (QCM)-based setup described in the experimental section.

Drop-cast films of the binary mixture (MOF + 10 wt % ODP) and the components alone (MOF or ODP) were prepared for comparison. The experiments were conducted at 303 K and a total pressure of 100 kPa, according to the procedure described in the experimental section.

Mass changes were calculated from frequency changes using the Sauerbrey equation [43], Δ*f* = −*C*_f_·Δ*m*, where Δ*f* corresponds to the observed frequency change in Hz, *C*_f_ is the sensitivity factor of the QCM crystal (0.1834 Hz·ng^−1^·cm^2^, provided by the manufacturer) and Δ*m* is the change in mass per unit area. The deposited mass of the film or drop-cast samples was determined by the change in the resonant frequency after the deposition. The adsorption of CO_2_ was determined from the frequency changes upon varying the CO_2_ content in the gas stream.

**Figure 3:**
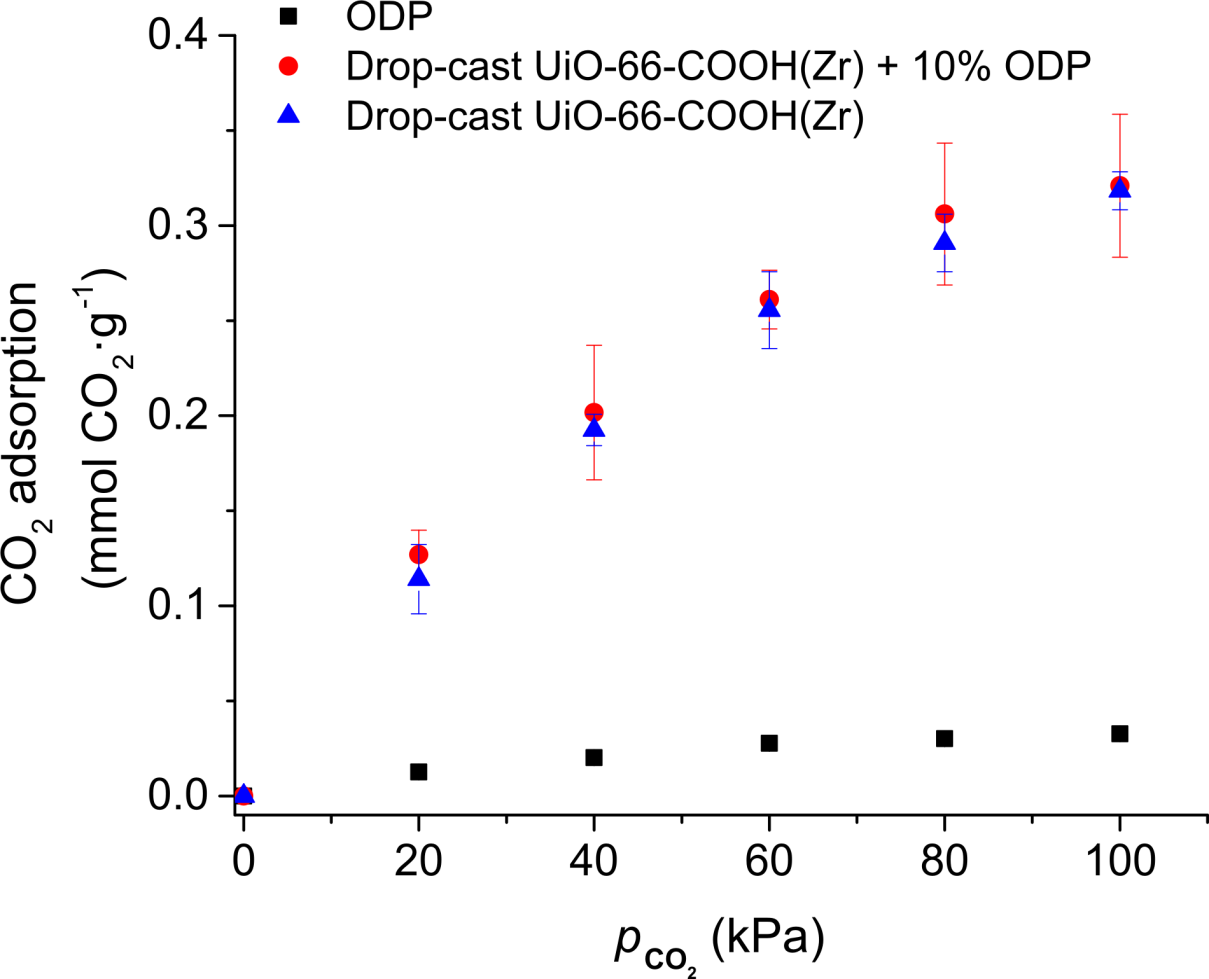
CO_2_ adsorption isotherms for drop-cast films: pure UiO-66-COOH(Zr) (blue triangles), pure ODP (black squares) and mixture UiO-66-COOH(Zr) + 10 wt % ODP (red circles).

Figure 3 shows CO_2_ adsorption isotherms obtained for drop-cast samples of pure UiO-66-COOH(Zr) sMPs and ODP and the mixture UiO-66-COOH(Zr) sMPs + 10 wt % ODP. The adsorption of ODP is almost negligible within the experimental error, while the adsorption of drop-cast films containing UiO-66-COOH(Zr) sMPs is lower than the value determined for the MOF powder using a conventional volumetric method. This has been also observed in previous studies with other MOF [26] and zinc imidazolate frameworks [44] and can be explained by the limitation of the MOF activation conditions in our experimental setup (80 °C and atmospheric pressure). It can also be observed that the CO_2_ adsorption capacity of the pure UiO-66-COOH(Zr) film is similar to that of mixed film UiO-66-COOH(Zr) + 10 wt % ODP, which is in good agreement with the results reported for other Zr-based MOFs that do not result in significantly reduced adsorption capacity after modification with ODP [34].

**Figure 4:**
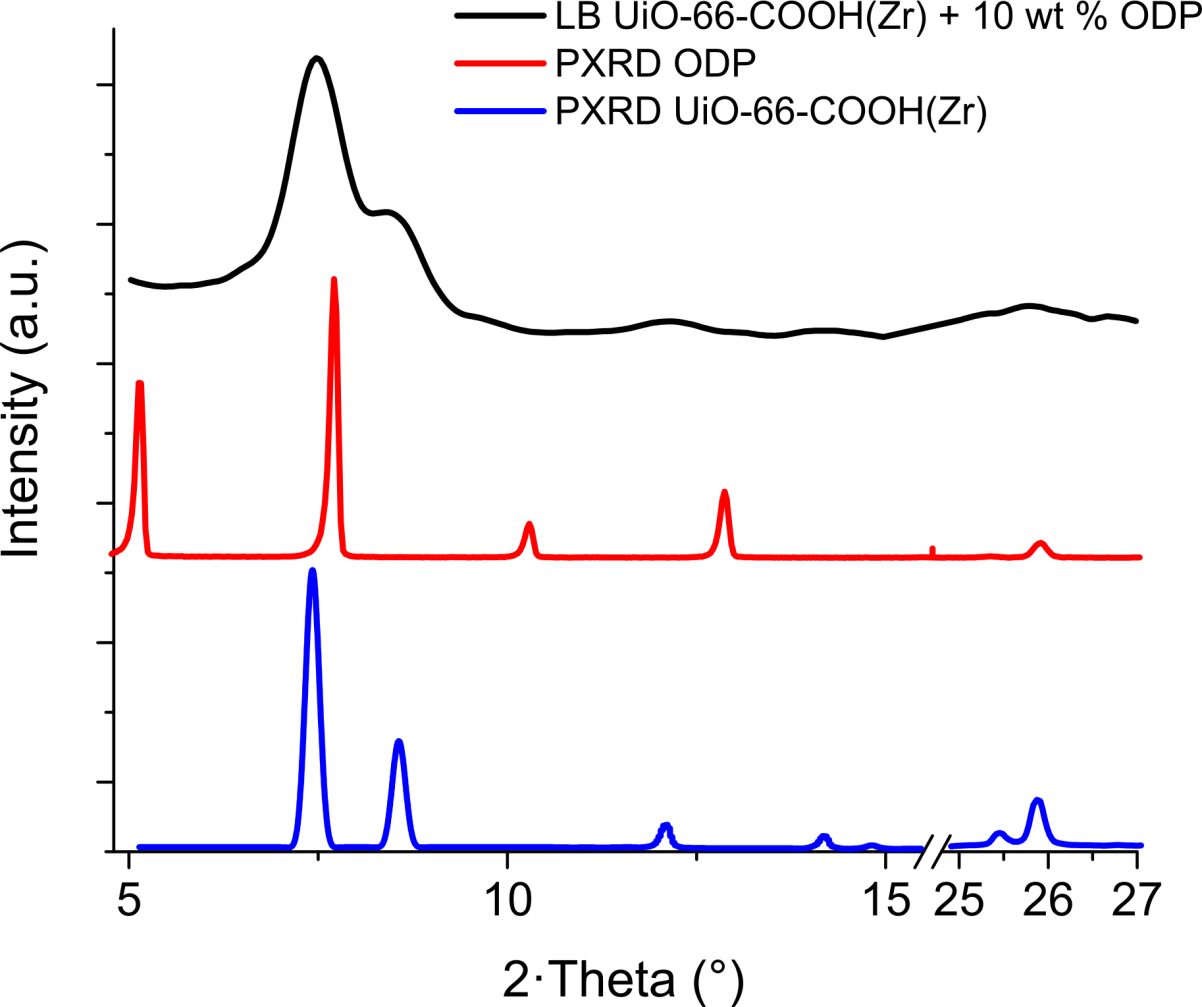
GIXRD pattern of an LB sample of UiO-66-COOH(Zr) + 10 wt % ODP (black line). For comparison purposes, the experimental powder diffraction of UiO-66-COOH(Zr) (PXRD, blue line) and ODP (PXRD, red line) is included.

To confirm that the addition of ODP does not alter the crystallinity of the MOF, grazing incidence X-ray diffraction (GIXRD) studies were performed both on LB and drop-cast samples deposited onto Si(100) substrates (Figure 4 and Supporting Information File 1, Figure S7). The GIXRD patterns of LB samples confirm the main peaks of UiO-66-COOH(Zr), at 7.4°, 8.5°, 12.1°, 25.5° and 25.9°, albeit broadened due to the reduced size of the particles. This broadening, together with the relatively lower intensity, results in some peaks appearing as unique broad humps (e.g., 14.1° and 14.8°, 25.5° and 25.9°). The ODP peaks are not visible on the LB GIXRD pattern due to the low ODP amount contained on the monolayer MOF/ODP film. In addition, a broad feature at 5.7° is present in the MOF/ODP drop-cast sample that corresponds to pure ODP (Supporting Information File 1, Figure S7). Therefore, the crystalline structure of the MOF is preserved upon incorporation of the ODP and film fabrication. FTIR was also used to confirm ODP incorporation in the LB films (Supporting Information File 1, Figure S8). C–H stretch bands from ODP (2920 and 2851 cm^−1^) are observed in the MOF/ODP LB films.

This demonstrates that this methodology is useful for modifying the surface of the particles without significantly affecting the porosity or crystallinity since both properties are intimately linked in a MOF. Finally, it should also be mentioned that the CO_2_ adsorption capacity at 1 bar for an LB film is similar to that of drop-cast samples but the relative deviations in the measurements of LB films were higher due to the low deposition of MOF (2 µg for the LB film and ≈6 µg for the drop-cast sample).

### Ultrathin hydrophobic coatings

To study the hydrophobic character of LB, LS and RLS films, water contact angle (WCA) values were measured on glass and mica substrates before and after coating with one monolayer mixed film MOF + 10 wt % ODP deposited at 30 mN·m^−1^. For comparison, LB and LS films of pure ODP transferred at the same surface pressure were also analyzed. The effect of MOF/ODP ultrathin coverage was similar in both hydrophilic substrates, independent of the transfer method, with WCA values in the range between 112° and 120°, compared to 10.1° and 40.8° for bare mica and glass, respectively (Table 1). These values considerably exceed the WCA obtained with NH_2_-MIL88B(Fe)/Matrimid^®^ on glass (66°), mica (19.6°), polysulfone (69°) and PIM-1 (74.5°) [28].

**Table 1:** Water contact angle values (average ± standard deviation) for ultrathin films transferred at 30 mN·m^−1^ using different transfer procedures (vertical: LB, horizontal: LS and RLS). The films were prepared using suspensions containing 0.05 mg·mL^−1^ of UiO-66-COOH(Zr) + 10 wt % ODP. For comparison purposes, uncoated substrates, LB and LS films of pure ODP transferred at 30 mN·m^−1^ and drop-cast films were also analyzed.

Sample	Substrate	WCA (°)	WCA (°) 12 months after film preparation

Uncoated substrate	glass	40.8 ± 0.3	^a^
	mica	10.1 ± 0.2	^a^
LB film MOF + 10 wt % ODP	glass	114.7 ± 0.6	107.2 ± 0.6
	mica	112.5 ± 0.4	105.9 ± 1.5
LS film MOF + 10 wt % ODP	glass	119.5 ± 0.8	120.2 ± 0.7
	mica	109.7 ± 1.6	^b^
RLS film MOF + 10 wt % ODP	glass	117.1 ± 0.3	110.5 ± 0.8
	mica	114.2 ± 0.8	106.0 ± 1.2
Drop-cast film MOF + 10 wt % ODP	glass	35.8 ± 0.8	^b^
	mica	20.1 ± 0.7	^b^
LB film ODP	glass	96.1 ± 0.5	99.4 ± 0.5
	mica	96.6 ± 2.4	92.8 ± 0.8
LS film ODP	glass	92.5 ± 0.8	85.4 ± 1.5
	mica	96.1 ± 0.3	92.3 ± 0.7

^a^Not relevant. ^b^Sample not available after 12 months.

Interestingly, even a highly hydrophilic substrate like mica showed a clear hydrophobic behavior after being coated with just one MOF/ODP film. Moreover, the increment of the WCA was significantly higher using MOF/ODP coatings than pure ODP films (WCA values between 92° and 99°) and deviations from the average WCA values were lower with mixed films, which seem to indicate that the coverage obtained with MOF/ODP films is more homogeneous than with pure ODP. Additionally, cast films of the mixture MOF/ODP were also prepared and characterized for comparison purposes, showing that the increment of the WCA is almost negligible (35.8° and 20.1° on glass and mica, respectively, see Table 1) if the films deposited do not have an ordered structure that completely covers the substrate’s surface. Finally, WCA values have been also measured 12 months after film preparation (Table 1), showing that the ultrathin coverages obtained in this contribution present a remarkable stability: mixed MOF + ODP ultrathin films present, in general, a decrease of the WCA of only ≈7° after this period, while LS films deposited on glass even show a slight increase of the WCA value.

Comparing these values to previous studies reported in the literature (see Table 2), it can be concluded that the films fabricated in this work are the thinnest coatings based on MOF showing hydrophobic properties reported up to date. Moreover, these hydrophobic ultrathin films show a high transparency (see Supporting Information File 1, Figure S9) which is an important characteristic in many practical applications.

**Table 2:** Comparative analysis of contact angles of hydrophobic MOFs reported in the literature.

MOF	Sample analyzed	WCA (°)	Ref.

UiO-66-COOH(Zr)	LS film MOF + 10 wt % ODP (thickness ≈200 nm) on glass	119.5 ± 0.8	this work
SIM-2	MOF film (thickness ≈20 µm) on Al_2_O_3_	>150	[37]
PCN-222	MOF film (thickness ≈100 µm) on glass	142	[45]
UHMOF-100	MOF@PDMS cross-linked film on PP fabric	135	[46]
NMOF-1	MOF film on glass	160–162	[47]
oCB-MOF-1	MOF packed on glass	140	[48]
MOFF-2	MOF powder dried in a vacuum oven	151 ± 1	[49]
MOFF-3	MOF powder dried in a vacuum oven	134 ± 1	[49]
MIL-53(Al)-AM4	MOF powder pressed onto glass with a spatula	>150	[50]
MIL-53(Al)-AM6	MOF powder pressed onto glass with a spatula	>150	[50]
OPA-UiO-66	MOF powder	160	[34]
Fluorinated ZIF-90	MOF powder	152.4	[51]
SH ZIF-67	MOF powder	146	[52]
UPC-21	MOF powder	145	[53]
Cu_3_(NH-AM10-BTC)_2_	MOF powder pressed on glass (height ≈2 mm)	147	[54]
PESD-1	MOF powder degassed (<10 µm)	>150	[36]
ZIF-8-VF	pressed MOF pellet	173	[55]

Aguado et al. [37] obtained hydrophobic SIM-2 by post-synthetic functionalization of SIM-1 films deposited onto anodic alumina disks (MOF layer thickness ≈20 µm). Grzybowski et al. [45] obtained freestanding, porphyrin-based MOF films approximately 100 µm-thick. Ghosh et al. [46] synthesized cross-linked structures formed by the fluorinated UHMOF-100 and the polymer PDMS that were coated onto polypropylene (PP) fabric by a spray coating technique (thickness of the coating not specified). Maji et al. [47] synthesized the three-dimensional supramolecular porous frameworks (NMOF-1) by coordination directed self-assembly of hydrophobic alkyl chains (OPE-C18) with Zn(II) and coated glass substrates using its ethanolic dispersions (thickness of the coating not specified). However, WCA values in most of the previous studies [34,36,48–55] have been measured using MOF powders or pellets and the use of these materials as coatings has not been optimized. In this contribution, the substrates have been coated with just one monolayer of mixed MOF/ODP films. Besides, these films contain a well-defined density of uniformly distributed MOF sMPs responsible for the highly hydrophobic character observed. Considering that the WCA values obtained with LB, LS and RLS films were similar, LB films were further characterized due to the advantageous automation of the transfer process in the Langmuir trough used in this study.

**Figure 5:**
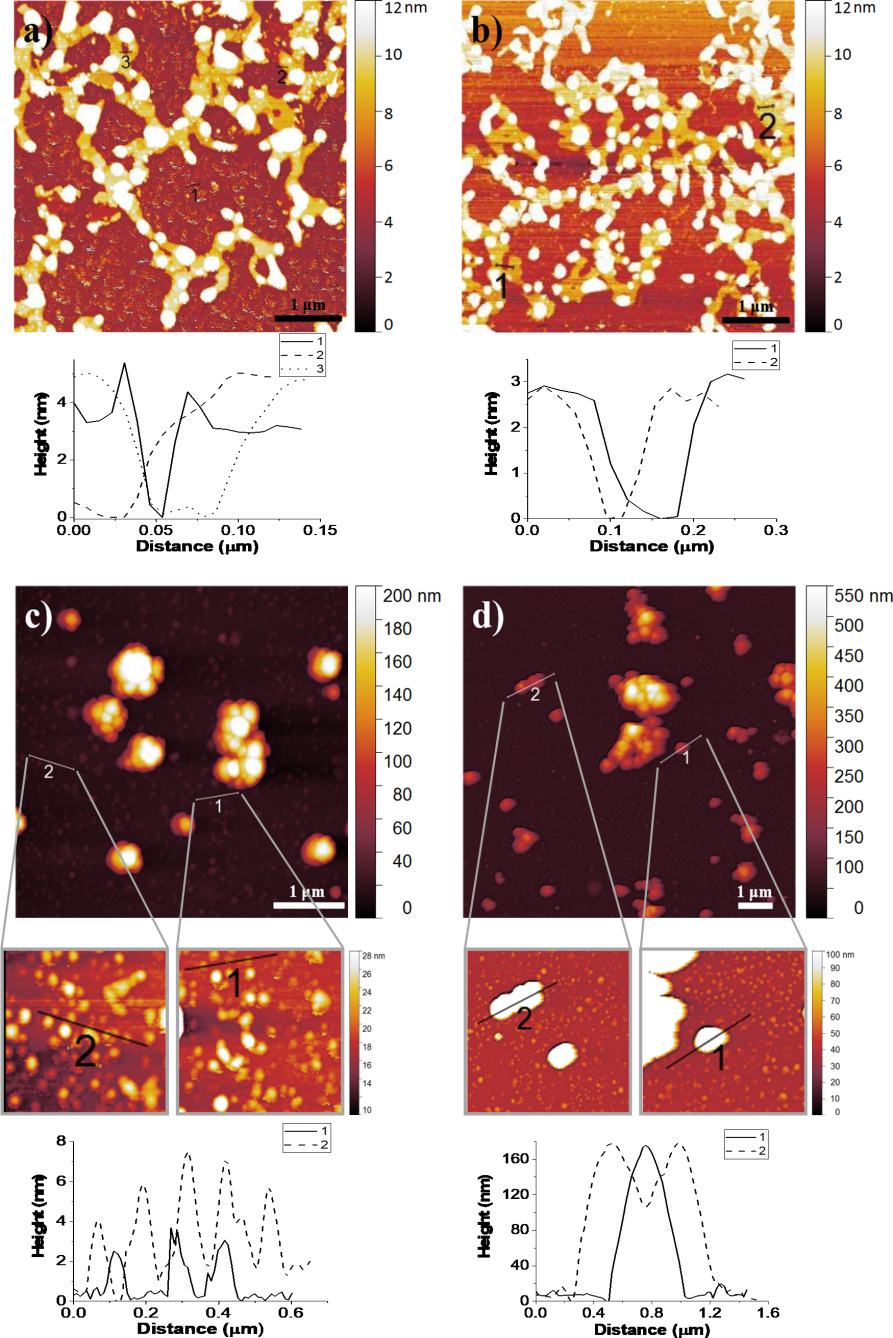
AFM images of a pure ODP LB film (a,b) and a mixed MOF/ODP LB film (c,d), transferred onto mica substrates at a surface pressure of 30 mN·m^−1^. RMS values of the images are: (a) 5.70 nm, (b) 6.41 nm, (c) 40.25 nm, (d) 61.28 nm. The films were prepared using suspensions containing 0.05 mg·mL^−1^ of UiO-66-COOH(Zr) + 10 wt % ODP.

To further investigate the structure of the hydrophobic films obtained, pure ODP LB films and mixed MOF/ODP LB films transferred onto mica were analyzed by AFM (Figure 5). The study of pure ODP monolayers showed that the films present some defects and pinholes, which allow a monolayer thickness between 2 and 3 nm to be determined. This value is in good agreement with the thickness previously reported for ODP monolayers deposited onto silver or gold substrates [33] and reveals that molecules in the film adopt an almost vertical position. Moreover, some domains of different sizes and heights up to 25 nm can be observed, which reveal higher accumulation of material. This was probably formed during film transfer, since the area per molecule at the surface pressure during transfer (0.2 nm^2^ per molecule) is smaller than the values obtained for self-assembled monolayers (0.25 nm^2^) of ODP onto mica [56].

The characteristic structure of the pure ODP films seems to be preserved in mixed MOF/ODP films and, in addition, MOF sMPs can be also observed. The presence of MOF particles significantly increases the roughness of the film (pure ODP LB film RMS values are close to 6 nm, while mixed films present RMS values between 40 and 60 nm), which leads to higher WCA values and confirms the advantageous interaction of ODP molecules with the surface of MOF sMPs. In fact, the phase images obtained by AFM, which show ODP covering the MOF sMPs (Supporting Information File 1, Figure S10), support this suggested synergy in order to obtain ultrathin hydrophobic coatings. The results obtained in this contribution suggest that the controlled coating of MOF sMPs can help to engineer surfaces at the nanoscale by finding synergy between surfactants and MOFs. Thus the use of LB, LS and RLS films can be an alternative to tools based on lithography that are common, for example, in the electronics industry.

## Conclusion

The formation of ultrathin films containing sMPs (size 200 ± 80 nm) of the metal organic framework UiO-66-COOH(Zr) at the air–water interface has been studied. Different solvents have been tested in order to improve the quality of the MOF dispersions and the spreading process. However, bare MOF films do not completely cover the water surface, probably due to the dissolution of MOF sMPs into the aqueous subphase.

As an alternative, the fabrication of mixed MOF/surfactant films has been optimized by using octadecylphosphonic acid. The addition of 10 wt % of ODP (relative to MOF mass) to MOF suspensions allows compact MOF/ODP monolayers at the air–water interface to be obtained without significant particle aggregation. These films can be deposited using the horizontal (LS and RLS) or vertical (LB) deposition methods onto hydrophilic substrates such as glass and mica and the coverage with just one mixed MOF/ODP layer increases the water contact angle up to 120°. The structure of these highly hydrophobic films has been characterized by GIXRD, FTIR, AFM and SEM, revealing that ODP forms a continuous film, with ODP molecules in an almost vertical position, and MOF sMPs, covered by ODP molecules, distributed over the whole surface, providing an elevated roughness to the mixed films that significantly contributes to increase the value of the water contact angle. Moreover, the crystallinity of the MOF particles is preserved in MOF/ODP mixed films.

Additionally, the CO_2_ adsorption capacity of bare UiO-66-COOH(Zr) cast films is similar to that of mixed UiO-66-COOH(Zr)/ODP films, which reveals that the mixture with ODP allows modification of the surface of the sMPs without significantly affecting its porosity.

Compared to other methodologies used for MOF modification in order to obtain highly hydrophobic materials, the fabrication of mixed films containing MOF sMPs and appropriate surfactants could be a very interesting alternative for the development of ultrathin coverage with elevated roughness that significantly increases the hydrophobicity of highly hydrophilic substrates.

## Experimental

### MOF synthesis and characterization

UiO-66-COOH(Zr) was synthesized following a previously reported protocol with slightly modified conditions [31]. 1,2,4-Benzenetricarboxylic acid (BTC) (2.2 g, 10 mmol) from Alfa Aesar (98%) was dissolved under stirring in 25 mL of distilled water at room temperature followed by the addition of zirconium tetrachloride (1.2 g, 5 mmol) from Alfa Aesar (>99.5%). The mixture was then heated under reflux (around 100 °C) for 24 h. The obtained white dispersion was filtered and washed with distilled water. Then, the solid was dispersed in ≈80 mL of distilled water. After heating the solution under reflux for 16 h, the mixture was filtered and washed with distilled water. Approximately 2 g of solid material was obtained from which one part was left to dry at ambient air and the remaining part was suspended in tetrahydrofuran (THF).

Powder X-ray diffraction data (PXRD) of the MOF were collected with a conventional (θ–2θ) Siemens D5000 diffractometer using a Cu radiation source (average Kα radiation λ = 1.5418 Å). The PXRD patterns of ODP were collected with a D-Max Rigaku diffractometer using a Cu radiation source operated at 40 kV and 80 mA (average Kα radiation λ = 1.5418 Å).

The sorption measurements were performed at 77 K on a BEL Japan Belsorp Mini apparatus using N_2_ as the probing gas. The samples were outgassed at 50 °C under vacuum for 24 h (BEL Japan, Belsorp Prep).

Scanning electron microscopy (SEM) images were recorded on a JEOL JSM-7001F microscope. The samples were coated with a layer of gold.

### Langmuir, Langmuir–Blodgett and reverse Langmuir–Schaefer film fabrication and characterization

The experimental methodology is similar to that described in [21,26,28]. Langmuir film formation was studied in a commercial Langmuir Teflon trough (NIMA, Model 702) with a symmetrical double-barrier configuration and dimensions of 720 × 100 mm. This device was used to register surface pressure vs area (π–*A*) and Brewster angle microscopy (BAM) images. Another apparatus (KSV-NIMA, Model 2000-System 3) was used for the fabrication of Langmuir–Blodgett (LB), Langmuir–Schaefer (LS) and reverse Langmuir–Schaefer (RLS) films. This commercial trough with dimensions of 775 × 120 mm is also equipped with a symmetrical double-barrier system. Both troughs were kept inside closed cabinets in a clean room at constant temperature (20 ± 1 °C). The compression of the water surface was performed at a constant speed of 6 cm^2^·min^−1^. Ultrapure Milli-Q water (ρ = 18.2 MΩ·cm) was used as the subphase in all the experiments. The surface pressure was continuously registered in both devices using Wilhelmy balances with a filter paper plate. BAM images were obtained using a KSV NIMA Micro BAM, equipped with a red laser light source (50 mW, 659 nm) with a fixed incidence angle of 53.1°. The spatial resolution of this optical system in the water surface plane is 6 µm per pixel.

Chloroform (CHCl_3_, Macron, >99.8%) was used to prepare UiO-66-COOH(Zr) suspensions. Different concentrations were tested in the range 0.01–0.11 mg MOF·mL^−1^. Tetrahydrofuran (Sigma-Aldrich, ≥99.9%), ethanol (VWR, >99.8%) and methanol (Sigma-Aldrich, 99.8%) were also used to prepare bare MOF and mixed MOF/surfactant suspensions. The suspensions were prepared from dry powders using a Selecta Ultrasons 3000683 ultrasonic bath (30 min) and magnetic stirring at room temperature overnight. In all cases, the suspensions were also sonicated for 30 min before using them for Langmuir/Langmuir–Blodgett film preparation.

Langmuir–Blodgett films were fabricated onto solid substrates (glass and mica) by vertical dipping at a speed of 1 mm·min^−1^. Glass substrates were purchased from Labbox Labware (SLIU-010-50) and mica from Ted Pella (Grade V1, Prod. No. 56). In the cases specified, glass substrates were immersed into a solution of 1,1,1,3,3,3-hexamethyldisilazane (Sigma-Aldrich, 99.9%) for 24 h prior to the transfer process to make the glass surface hydrophobic. The substrates were then rinsed with chloroform to remove the excess silane. Unless otherwise stated, the transfer was performed during substrate emersion.

Langmuir–Schaefer films were also fabricated onto the same type of solid substrates used for Langmuir–Blodgett film fabrication. The substrate was held horizontal and parallel to the water surface using a plastic clamp. When the desired surface pressure was reached, the substrate was approached to the surface at a vertical speed of 1 mm·min^−1^. Once the substrate touched the water surface, it was withdrawn at a vertical speed of 10 mm·min^−1^. In the stated cases, a modified protocol was used. In the so-called reverse Langmuir–Schaefer (RLS) transfer, the substrate was held horizontal to the water surface but immersed in the subphase. The substrate was then slowly raised through the interface when the target surface pressure was achieved.

The drop-cast samples were fabricated spreading drop by drop a total volume of ≈150 µL on the top of the glass and mica substrates similar to those used in LB film fabrication and QCM disks. The suspensions containing 0.5 mg·mL^−1^ of pure MOF were used and a proper amount of ODP was added to prepare the mixture containing the MOF and 10 wt % of ODP.

SEM images were taken at 10 kV with a FEG column using a SEM Inspect F50 (FEI Company). All samples were coated with a layer of platinum (5–10 nm) prior to SEM inspection.

IR spectra were obtained using a Perking Elmer Spectrum 100 spectrometer operated in transmission mode for films and in attenuated total reflectance (ATR) mode for powder samples. The films were deposited onto calcium fluoride windows.

Water contact angle measurements were performed on different substrates using an optical tensiometer (Theta Lite) purchased from Attension. Average values and error are calculated from four measurements performed at different positions of each sample.

Atomic force microscopy (AFM) imaging was conducted on a NTEGRA Aura microscope from NT-MDT under ambient conditions. The equipment was operated in semicontact mode using a SF005&AU006NTF head. AFM data were collected using NT-MDT HA_NC(B) silicon tips with typical spring constant and resonant frequency of 3.5 N·m^−1^ and 140 kHz, respectively.

Grazing incidence X-ray diffraction (GIXRD) characterization of films deposited onto Si(100) wafers was done using a high-resolution Empyrean diffractometer (PANalytical) operating at 45 kV (generator voltage) and a tube current of 40 mA (Cu Kα radiation). A Pixcell 1D medipix3 detector operating in the open detector mode was used. An optimization of the grazing incidence angle was performed for each sample prior to scan acquisition and values were between 0.08° and 0.10°.

CO_2_ adsorption was measured using a homemade QCM-based system [21,26] to investigate the effect of ODP on the adsorption capacity of the sMPs. Briefly, the setup consists of two chlorinated polyvinyl chloride (CPVC) CHC-15 crystal holders from Inficon inside a stainless steel cell with a total volume about 200 mL. Two 9 MHz AT-cut QCM crystals (Inficon) are used in each experiment, where one uncoated disk is used as a reference to correct possible frequency drifts mainly due to temperature or gas flow. CO_2_ was used as an adsorbate and its concentration into the chamber was controlled with He as a diluting gas. A total gas flow of 50 mL (STP)·min^−1^ was used in all the experiments and each gas flow was controlled separately using an Alicat Scientific MC-100SCCM-D/5M mass-flow controller. Prior to each measurement an outgassing step was performed at 353 K for 2 h by flowing He at 50 mL (STP)·min^−1^. Then, the sample was cooled down to 303 K maintaining the same He flow until the frequency stabilizes. The adsorption measurements were conducted at constant temperature (303 K) and five different CO_2_ partial pressures were used corresponding to 20%, 40%, 60%, 80% and 100% of CO_2_ in volume. As a final step, pure He was used to observe the recovery of the frequency due to eventual CO_2_ removal.

## Supporting Information

PXRD, SEM and N_2_ sorption isotherm characterization of UiO-66-COOH(Zr) submicrometer particles. Description of the optimization process of Langmuir films using solvent mixtures and complementary surface pressure/area isotherms, BAM images, SEM images, GIXRD, FTIR and AFM characterization of UiO-66-COOH(Zr) + ODP Langmuir and Langmuir–Blodgett films and a picture comparing the transparency of a glass substrate covered with an LB monolayer MOF/ODP with an uncovered glass are also included.

File 1 Additional experimental data.
