# Supplementary material for: Ultrathin hydrophobic films based on the metal organic framework UiO-66-COOH(Zr)
Source: Beilstein J Nanotechnol. 2019 Mar 6;10:654–65. doi: 10.3762/bjnano.10.65 (PMC6423563; doi:10.3762/bjnano.10.65)
Supplement: File 1 — Additional experimental data. [file Beilstein_J_Nanotechnol-10-654-s001.pdf]

BEILSTEIN JOURNAL OF NANOTECHNOLOGY

Supporting Information

for

Ultrathin hydrophobic films based on the metal organic framework UiO-66-COOH(Zr)

Miguel A. Andrés, Clemence Sicard, Christian Serre, Olivier Roubeau and Ignacio Gascón

Beilstein J. Nanotechnol. **2019,** *10,* 654–665. doi:10.3762/bjnano.10.65

Additional experimental data

License and Terms: This is a supporting information file under the terms of the Creative Commons Attribution License (http://creativecommons.org/licenses/by/4.0). Please note that the reuse, redistribution and reproduction in particular requires that the authors and source are credited.
The license is subject to the *Beilstein Journal of Nanotechnology* terms and conditions: (https://www.beilstein-journals.org/bjnano)

Characterization of UiO-66-COOH(Zr) MOF submicronic particles

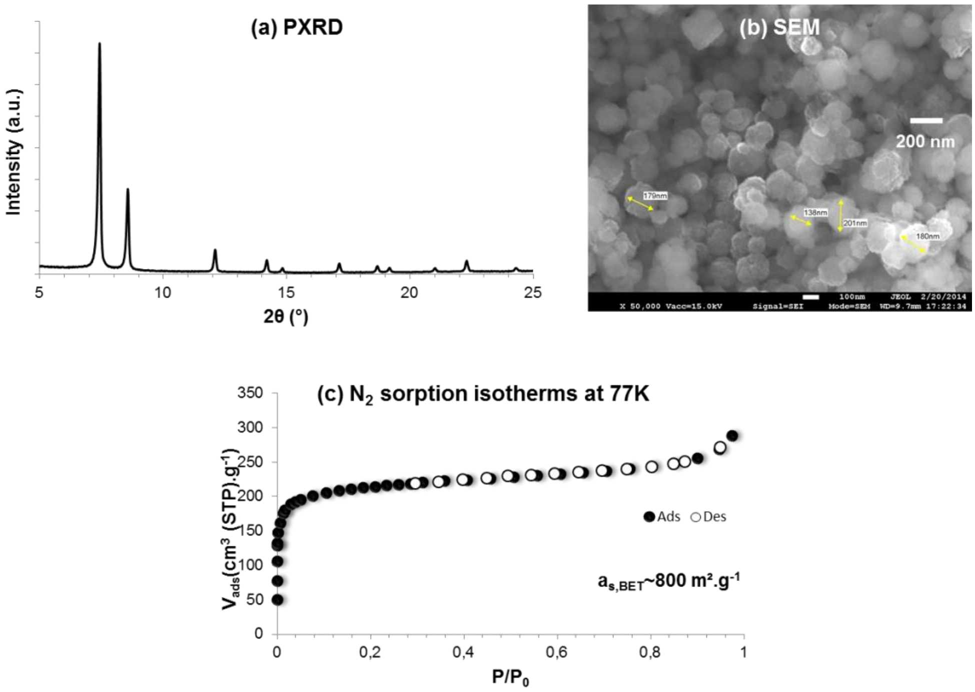

Figure S1: Characterization of UiO-66-COOH(Zr) submicronic particles. (a) Powder X-ray diffractogram; (b) Scanning electronic microscopy image; (c) N_2 sorption isotherms at 77 K with associated BET surface area of ~ 800 m^2/g.

Optimization of Langmuir films: solvent mixtures

Langmuir films of UiO-66-COOH(Zr) were obtained at the air-water interface using diluted MOF suspensions in chloroform (concentration range 0.01–0.05 $mg{\cdot}mL^{-1}$). Surface pressure-area (π–A) isotherms were registered spreading different volumes of each suspension to find the optimal volume for each MOF concentration. A lack of reproducibility was observed in all cases. To find an explanation to this phenomenon, the change in the isotherms along all the concentration range was compared. Figure S2 shows representative π–A isotherms for each MOF concentration assayed. Upon increasing MOF concentration, the lift-off of the isotherm appears at lower areas per MOF mass. This is normally reasoned by the fact that not all the theoretical MOF amount

(i.e., MOF mass multiplied by the spread volume) was in the air–water interface, either due to a bad suspension stability (i.e., a different MOF amount was spread) or by aggregation or dissolution into the water subphase.

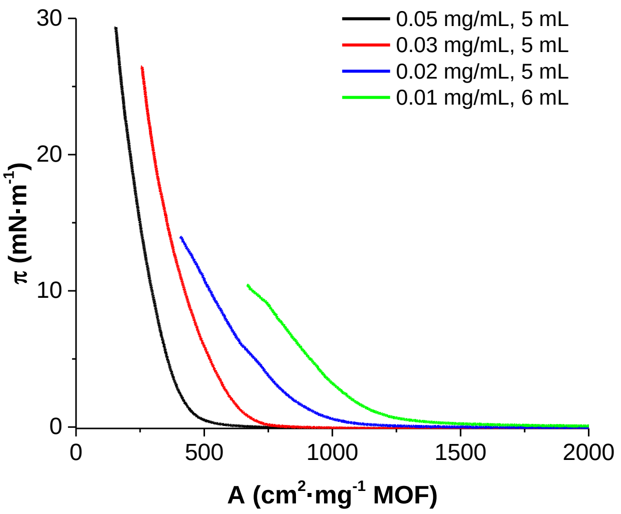

Figure S2: Surface pressure-area (π–*A*) isotherms obtained using different UiO-66-COOH(Zr) sMPs suspensions in chloroform, concentration range 0.01–0.05 mg·mL^{-1}.

To further analyze this behavior, the contact cross-sectional areas (CCSA) were linearly correlated to the theoretical spread amount of MOF. If the behavior of a given substance for Langmuir film formation is reproducible (e.g., no 3D aggregates), the adjustment of these variables should be approximately linear (i.e., an approximately equal CCSA per MOF mass) [1]. Figure S3 shows this adjustment. It is clearly seen that the tendency obtained with the more diluted suspensions is different than the behavior of the more concentrated ones. This can be explained by poorer suspension stability due to particle aggregation at higher concentrations. The presence of aggregates could be confirmed by BAM images even at high areas per mass of MOF (Figure S4). In addition, the

presence of free carboxylic acid groups in the framework could enhance the MOF dispersion into the aqueous subphase.

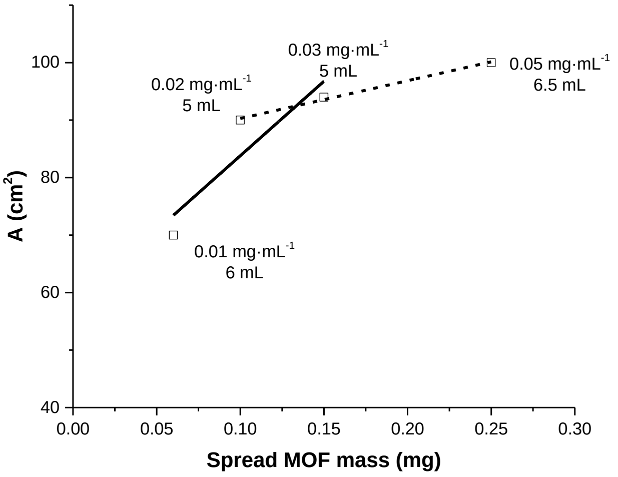

Figure S3: Linear correlation between CCSA and spread mass of MOF for four different UiO-66-COOH(Zr) suspensions in the concentration range 0.01–0.05 mg·mL^{-1}. Black and dashed lines correspond to the linear adjustment of points 0.01, 0.02 and 0.03 mg·mL^{-1} and 0.02, 0.03 and 0.05 mg·mL^{-1}, respectively.

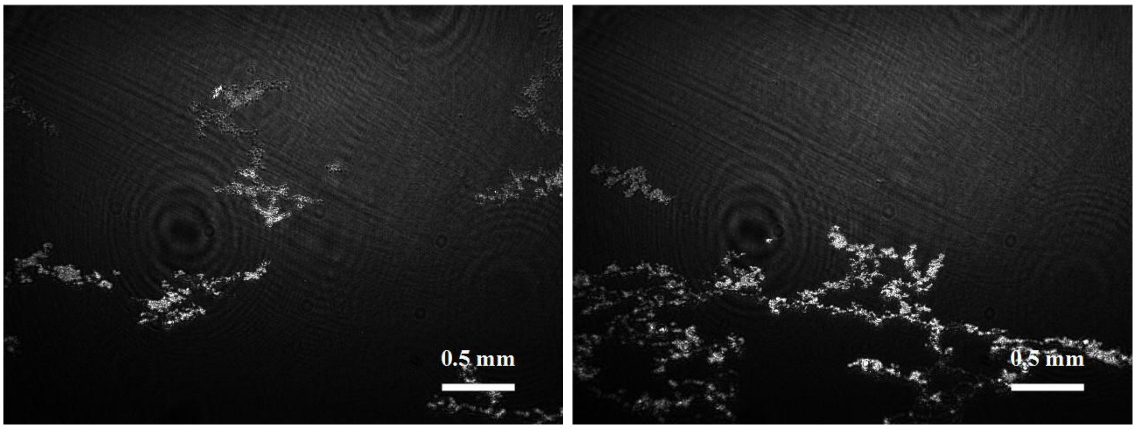

Figure S4: BAM images obtained at different areas per MOF mass using a 0.03 mg·mL^{-1} UiO-66-COOH(Zr) dispersion: (a) 7000.0 cm^2·g MOF^{-1}, (b) 5000.0 cm^2·g MOF^{-1}.

In order to improve the suspension stability and reduce the particle aggregation, the use of different solvent mixtures was explored. For these studies, other less hydrophobic solvents were mixed in different proportions with chloroform. The use of short chain alcohols (methanol and ethanol) with low boiling points was

first explored in order to improve the dispersibility of the MOF by hydrogen bonding and solvation by the interaction of the free pendant carboxylic group in the MOF and the hydroxyl group of the alcohol. Given the fact that these alcohols are miscible with water and can cause material loss into the subphase [2], alcohol/chloroform volume ratios not higher than 1:4 were tried. These MOF suspensions also showed poor stability. In addition, more compressed π–*A* isotherms were obtained and lower surface pressures were reached. Moreover, from BAM images a lower amount of MOF at the air–water interface was observed. Partial loss of the MOF into the subphase could explain these results. On views of these results, the use of other volatile solvents with a higher hydrophobicity than short chain alcohols was explored. THF was chosen as it is also used in previous studies for Langmuir film fabrication [1,3,4]. Different THF/$CHCl_3$ proportions in the range from 1:4 to pure THF were studied. In all cases, the dispersibility of MOF sMPs was greatly improved. In addition, the amount of precipitate after several hours without magnetic stirring was much lower in comparison to the suspensions in chloroform indicating an improvement in suspension stability. An expansion is observed in the π–*A* isotherms when increasing the THF content in the mixtures from a 1:4 to a 1:1 THF/$CHCl_3$ volume ratio. However, when the THF volume fraction in the mixtures is increased above 0.5, a compression in the normalized π–*A* isotherms is observed. The results suggest on the one hand an improvement in the dispersibility of the material with increasing THF content, in agreement with the enhanced stability of the suspensions which were stable overnight without mechanical stirring. On the other hand, a compression on the isotherms upon predominance of THF in the suspensions is in accordance with the loss of

material by dissolution into the subphase as THF is water-miscible. The better reproducibility was obtained with solvent mixtures containing a 1:4 $THF/CHCl_3$ volume ratio. These mixtures were further assayed looking for a compromise between the dispersibility of the material in the suspension and the miscibility of the suspension in the subphase. However, from the analysis of the surface pressure-area isotherms, some material loss occurred when the same nominal amount of MOF was spread using dispersions of different concentrations. The use of wet MOF instead of dry powder did not improve the dispersibility or Langmuir film formation in any of the solvents studied.

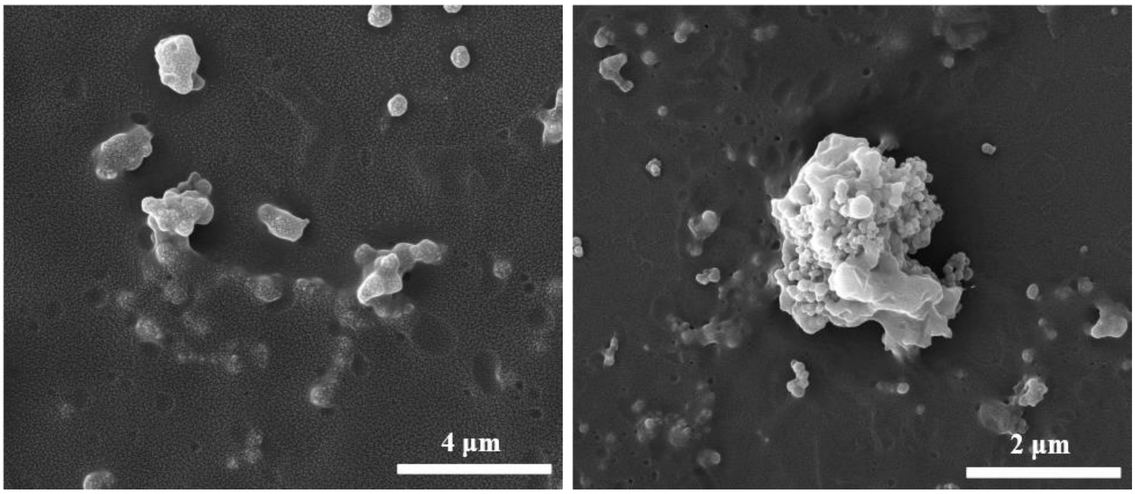

Figure S5: SEM images of a drop-cast sample from a UiO-66-COOH(Zr) + 10 wt % behenic acid dispersion showing loss of particle morphology due to degradation or reaction of the MOF particles with the surfactant.

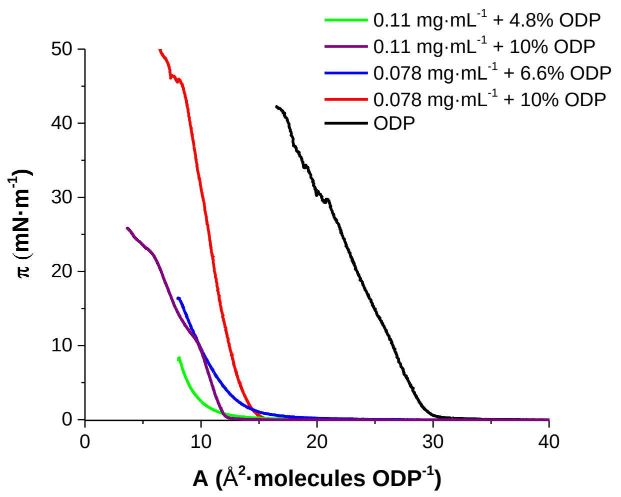

Figure S6: Surface pressure-area isotherms (π–*A*) obtained using different UiO-66-COOH(Zr) + 10 wt % ODP dispersions in the MOF concentration range 0.078–0.11 mg·mL^{-1}. For comparison purposes, isotherms for the same MOF concentration but ODP content of 0.8 μmol·mL^{-1} are included. Note that area per ODP molecule is represented to compare the effect of different surfactant concentration.

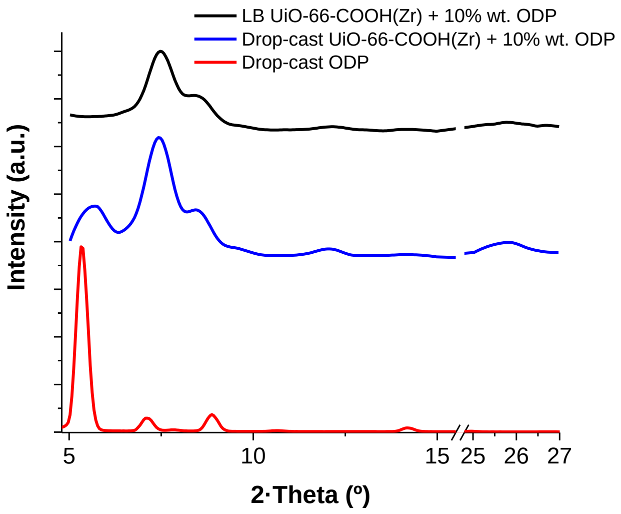

Figure S7: GIXRD patterns of drop-cast (blue) and LB (black) samples of UiO-66-COOH(Zr) + 10 wt % ODP. For comparison purposes, drop-cast of pure ODP (red) is included.

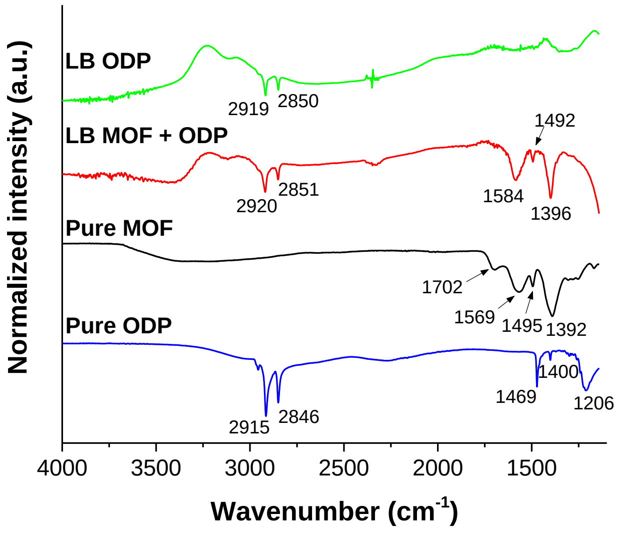

Figure S8: FTIR spectra of ODP and MOF/ODP LB films deposited onto calcium fluoride substrates. For comparison purposes, FTIR-ATR spectra of pure MOF and ODP are included.

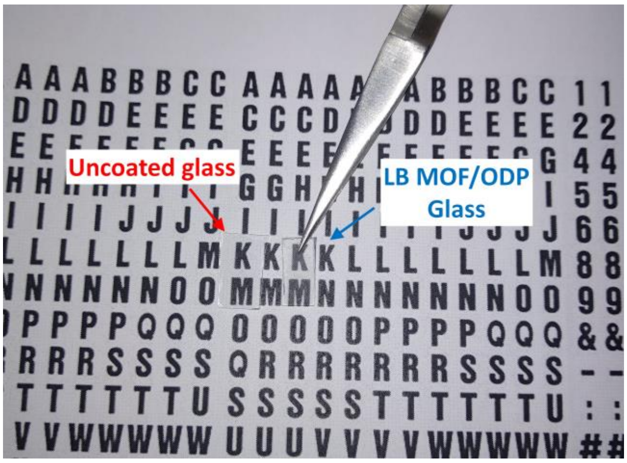

Figure S9: Comparison of the transparency of a glass substrate covered with an LB monolayer MOF/ODP with an uncovered glass.

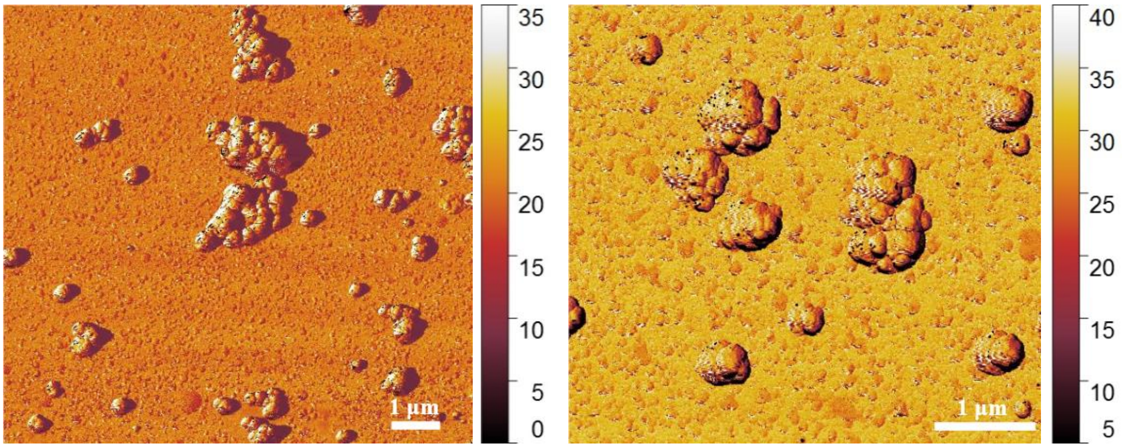

Figure S10: Phase images of mixed MOF/ODP LB films, transferred onto mica substrates at a surface pressure of 30 mN·m^{-1} showing ODP coverage of MOF sMPs. Suspensions containing 0.05 mg·mL^{-1} of UiO-66-COOH(Zr) + 10 wt %. ODP were used to prepare the LB films.

References

1. Paczesny, J.; Wolska-Pietkiewicz, M.; Binkiewicz, I.; Wadowska, M.; Wróbel, Z.; Matuła, K.; Nogala, W.; Lewiński, J.; Hołyst, R. *ACS Appl. Mater. Interfaces* **2016,** *8,* 13532–13541. doi:10.1021/acsami.6b03579

2. Gericke, A.; Simon-Kutscher, J.; Huehnerfuss, H. *Langmuir* **1993,** *9,* 2119–2127. doi:10.1021/la00032a036

3. Schöne, A.-C.; Schulz, B.; Richau, K.; Kratz, K.; Lendlein, A. *Macromol. Chem. Phys.* **2014,** *215,* 2437–2445. doi:10.1002/macp.201400377

4. Rubira, R. J. G.; Aoki, P. H. B.; Constantino, C. J. L.; Alessio, P. *Appl. Surf. Sci.* **2017,** *416,* 482–491. doi:10.1016/j.apsusc.2017.04.155
